# Patch testing in Iranian children with allergic contact dermatitis

**DOI:** 10.1186/s12895-016-0047-0

**Published:** 2016-07-12

**Authors:** Hossein Mortazavi, Amirhooshang Ehsani, Seyed Sajed Sajjadi, Nessa Aghazadeh, Ebrahim Arian

**Affiliations:** Autoimmune Bullous Diseases Research Center, Razi Dermatology Hospital, Tehran University of Medical Sciences, Tehran, Iran; Razi Dermtology Hospital, Tehran University of Medical Sciences, Tehran, Iran; Children’s Medical Hospital, Tehran University of Medical Sciences, Tehran, Iran; Sharif University of Technology, Tehran, Iran

**Keywords:** Allergic contact dermatitis, Patch testing, Children, Adolescent, Contact allergens

## Abstract

**Background:**

Allergic contact dermatitis is a common disorder in adults and children alike and appears to be on the increase. The purpose of this study was to determine the sensitization trends in Iranian children with contact dermatitis.

**Methods:**

The result of 109 patch tests performed using the 24 allergens of the European Standard Series in patients below 18 years old from September 2007 to March 2009 were recorded and analyzed. The tests were evaluated at 48 and 72 h after performing.

**Results:**

The study population consisted of 72 (66.1 %) females and 37 (33.9 %) males. Hands were the most commonly affected anatomic site. In the final evaluation of the tests on day three, 51 (46.8 %) individuals showed a positive reaction to at least one allergen. Females were significantly more likely to show a positive response to at least one allergen (*p*-value = 0.031, odds ratio: 2.46). The most common allergens were nickel sulfate, cobalt, methylisothiazolinone, and colophony with 21 (19.3 %), 11 (10.1 %), 7 (6.4 %), and 6 (5.5 %) positive reactions, respectively. Contact allergy to nickel sulfate was more common in females than males (23.6 % vs. 10.8 %). There was no statistically significant relationship between personal or family history of atopy and a positive reaction to patch testing. The clinical and practical relevance were assessed for nickel and cobalt with a clinical current relevance in 11 (52.3 %) and 4 (36.4 %), respectively.

**Conclusions:**

Nickel sulfate, cobalt, methylisothiazolinone, and colophony are the most common allergens responsible for induction of allergic contact dermatitis in Iranian children and adolescents. Females tended to show more positive reactions to allergens.

**Electronic supplementary material:**

The online version of this article (doi:10.1186/s12895-016-0047-0) contains supplementary material, which is available to authorized users.

## Background

Allergic contact dermatitis (ACD) is an inflammatory skin disease caused by a T-cell-mediated delayed-type hypersensitivity reaction [1]. In ACD, the hapten is initially introduced to the epidermal langerhans cells. These cells migrate to the regional lymph nodes and the allergen is subsequently processed by the T-lymphocytes. Upon re-exposure of the allergen, CD8+ T-cells response is mediated by the CD4+ T-cell subset [2].

ACD affects up to 20 % of the pediatric population [3]. According to previous studies, 14.5 to 70.7 % of children with a clinical diagnosis of contact dermatitis have positive reactions to one of the applied allergens for patch testing [4, 5]. Based on a recent review of five patch test studies in children, the most commonly reported allergens in children are neomycin, balsam of Peru, fragrance mix, lanolin, cocamidopropylbetaine, formaldehyde, corticosteroids, methylchlorisothiazolinone/methylisothiazolinone, propylene glycol, and benzalkonium chloride [6]. Properly performed and interpreted patch testing is the gold standard for identification and documentation of allergic sensitization and its inducing agents in children and adults [7, 8]. Although patch testing in children is not approved by the FDA, it has proven to be a safe procedure both in adults and children [9]. There are no rules and limitations for patch testing in children in Iran. Therefore, we often use it on similar approved indication for adults.

The purpose of this study was to determine the causes of allergic contact dermatitis and identify the pattern of allergen responsiveness in Iranian children and adolescents affected by ACD.

## Methods

The study was approved by Tehran University of Medical Scienced board of ethics. Registered data of 109 patch tests performed in individuals younger than 18 years old diagnosed clinically and/or histopathologically with allergic contact dermatitis and referred by dermatologists for patch testing were collected and analyzed. We used the ESS (European Standard Series Hermal, Reinbek, Germany) at similar allergen concentrations as for adults. The allergens were applied on the healthy skin of the patients’ backs and left for 48 h. Readings were performed at 48 h, 72 h and after patch testing (on day 2 and day 3). Reactions were classified and documented according to the criteria of the International Contact Dermatitis Research Group (ICDRG) as follows: (0 = negative), (+/− = doubtful), (+ = erythema), (++ = papule vesicle formation), and (+++ = bulla formation or ulceration) [7].

### Statistical analysis

Data management and descriptive statistical analysis were performed using the SPSS 16 statistical program. Chi-square and Fisher’s exact tests were used and a *P-value* less than 0.05 was considered a statistically significant difference.

## Results

The study population consisted of 37 (33.9 %) males and 72 (66.1 %) females. The mean age was 14.4 with a standard deviation of 3.4 years (range: 5–18 years). A positive personal history of atopy was recorded in 48 patients (44.0 %). Forty one (37.6 %) had a positive family history of atopy. Anatomic sites of involvement in order of frequency are shown in Table 1. Hands were the most common site of involvement (Additional files 1 and 2).

**Table 1:** Anatomic sites of involvement in order of frequency

Anatomic sites of involvement	Frequency
Hand	84 (77.1 %)
Face	30 (27.5 %)
Calf	23 (21.1 %)
Foot	17 (15.6 %)
Back	9 (8.2 %)
Forearm	9 (8.2 %)
Abdomen	8 (8.7 %)
Knee	7 (7.6 %)
Thorax	7 (7.6 %)
Arm	6 (6.5 %)
Body	6 (6.5 %)

Results of patch test reading on day 2 and re-evaluation on day 3 (72 h) with regard to one or more reaction to allergens or negative or doubtful results in male and female patients are shown in Table 2.

**Table 2:** The overall positive, doubtful and negative responses to patch test allergens among male and female patients

Gender	Positive	Percent	Negative	Percent	Doubtful	Percent
Male (*N* = 37)	12	32.4	12	32.4	13	35.2
Female (*N* = 72)	39	54.2	25	34.7	8	11.1
Total (*N* = 109)	51	46.8	37	33.9	21	19.3

Females were significantly more likely to show a positive response to at least one allergen (*p*-value = 0.031, odds ratio: 2.46, 95 %, and confidence interval (CI): 1.07–5.64). We found no significant difference in the frequency of positive patch test responses among patients with and without personal or family history of atopy (Table 3).

**Table 3:** Comparison of reactions to allergens (at least one positive or negative reaction) in patients with or without personal or family history of atopy

		Total	Reaction to allergens		*p*-value
			Positive (one or more reactions)	Negative	
personal history of atopy	+	48	26 (54.2 %)	22 (45.8 %)	0.171
	-	61	25 (41.0 %)	36 (59.0 %)	
family history of atopy	+	41	23 (56.1 %)	18 (43.9 %)	0.130
	-	68	28 (41.2 %)	40 (58.8 %)	

The overall results of patch test reading on day 2 and re-evaluation on day 3 based on gender are shown in Table 4. The ten most common inciting allergen in our series were nickel sulfate (21,19.3 %), cobalt chloride (11,10.1 %), methylisothiazolinone (7,6.4 %), colophony (6,5.5 %), potassium dichromate (5,4.6 %), paraben mix (4,3.7 %), 4-tert-butylphenon (4,3.7 %), fragrance mix (4,3.7 %), thiuram mix (3,2.8 %), mercapto mix (3,2.8 %), 4-phenylendiamine base (2,1.8 %), and formaldehyde (2,1.8 %) (Additional file 2).

**Table 4:** Positive response to allergens of the European standard series in Iranian children with allergic contact dermatitis

Substance	Concentration (%)	Total (*N* = 109)	Percent	Male (*N* = 37)	Percent	Female (*N* = 72)	Percent	*P-Value*	Relevance
Nickel sulfate	5	21	19.3	4	10.8	17	23.6	0.130	11 (52.3 %)
Cobalt chloride	1	11	10.1	5	13.5	6	8.3	0.504	4 (36.4 %)
Methylisothiazolinone	0.05	7	6.4	1	2.7	6	8.3	0.419	NA
Colophony	20	6	5.5	1	2.7	5	6.9	0.662	NA
Potassium dichromate	0.5	5	4.6	3	8.1	2	2.8	0.334	NA
Paraben mix	16	4	3.7	2	5.4	2	2.8	0.603	NA
4-tert-butylphenol formaldehyde resin	1	4	3.7	0	0.0	4	5.6	0.297	NA
Fragrance mix	8	4	3.7	1	2.7	3	4.2	1.000	NA
Thiuram mix	1	3	2.8	1	2.7	2	2.8	1.000	NA
Mercapto mix	1	3	2.8	0	0.0	3	4.2	0.550	NA
4-Phenylenediamine base	1	2	1.8	0	0.0	2	2.8	0.547	NA
Formaldehyde	1	2	1.8	1	2.7	1	1.4	1.000	NA
N-isopropyl-N-phenyl-4-phenylenediamine	0.1	2	1.8	1	2.7	1	1.4	1.000	NA
Wool Alcohol	30	2	1.8	0	0.0	2	2.8	0.547	NA
Sesquiterpene lactone mix	0.1	2	1.8	2	5.4	0	0.0	0.113	NA
Benzocaine	5	2	1.8	0	0.0	2	2.8	0.547	NA
Cliquinol	5	1	0.9	1	2.7	0	0.0	0.339	NA
Balsam of peru	25	1	0.9	0	0.0	1	1.4	1.000	NA
Epoxy resin	1	1	0.9	0	0.0	1	1.4	1.000	NA
Quaterium-15	1	1	0.9	0	0.0	1	1.4	1.000	NA
Mercaptobenzothiazole	2	1	0.9	1	2.7	0	0.0	0.339	NA
Neomycin sulphate	20	1	0.9	0	0.0	1	1.4	1.000	NA
Hydroxyl-methyl-penthyl-cyclo-carboxaldehyde	5	1	0.9	1	2.7	0	0.0	0.339	NA
Primin	0.01	0	0.0	0	0.0	0	0.0	NA	NA

Positive allergic reaction to nickel was more common in females than in males, however, without statistical significance (23.6 % vs. 10.8 %, *p*-value = 0.130) (Table 4).

We could only assess the clinical and practical relevance in the two most important allergens (nickel and cobalt), and we observed clinical current relevance in 11 (52.3 %) and 4 (36.4 %) for nickel and cobalt, respectively.

Results of patch test reading according to age groups are shown in Table 5.

**Table 5:** The positive patch test responses according to the inciting allergen and age group (0–10, 11–8 years)

Substance	0–10 (*N* = 22)	Percent	11–18 (*N* = 87)	Percent	*P-Value*
Nickel sulfate	3	13.6	18	20.7	0.55
Cobalt chloride	4	18.2	7	8.0	0.22
Methylisothiazolinone	2	9.1	5	5.7	0.62
Colophony	1	4.5	5	5.7	1.0
Potassium dichromate	0	0.0	5	5.7	0.58
Paraben mix	2	9.1	2	2.3	0.17
4-tert-butylphenon	0	0.0	4	4.6	0.58
Fragrance mix	1	4.5	3	3.4	1.0
Thiuram mix	0	0.0	3	3.4	1.0
Mercapto mix	0	0.0	3	3.4	1.0
Phenylendiamine base	0	0.0	2	2.3	1.0
Formaldehyde	0	0.0	2	2.3	1.0
N-isopropyl-N-phenyl-4-phenylenediamine	0	0.0	2	2.3	1.0
Wool Alcohol	0	0.0	2	2.3	1.0
Sesquiterpene lactone mix	0	0.0	2	2.3	1.0
Benzocaine	0	0.0	2	2.3	1.0
Cliquinol	0	0.0	1	1.1	1.0
Balsam of peru	0	0.0	1	1.1	1.0
Epoxy resin	0	0.0	1	1.1	1.0
Quaterium-18	0	0.0	1	1.1	1.0
Mercaptobenzothiazole	0	0.0	1	1.1	1.0
Neomycin sulphate	0	0.0	1	1.1	1.0
Hydroxyl-methyl-penthyl-cyclo-carboxaldehyde	0	0.0	1	1.1	1.0
Primin	0	0.0	0	0.0	NA

For most allergens (except cobalt chloride, methylisothiazolinone, paraben mix, and fragrance mix), the percentages of positive response in the older age group (11–18 years) were higher than children below 10 years.

The most common allergen in the younger age group were cobalt chloride (4, 18.2 %), nickel sulfate (3, 13.6 %), methylisothiazolinone (2, 9.1 %), and paraben mix (2, 9.1 %) while in the older group they were as follows: nickel sulfate, cobalt chloride, methylisothiazolinone, colophony, potassium dichromate.

## Discussion

In this study we have identified the inciting allergen according to patch tests in 109 children with ACD. Contact dermatitis in children has been studied less extensively than adults in the existing literature [10, 11].

In the current study, overall 48.6 % of patients had one or more positive patch test results. The positive response rate to patch test allergens ranges from 15 to 62.3 % in different studies [5, 12, 13].

Nickel was the most common allergen in our study, a common finding with most previous reports [10, 14, 15]. Although some authors have reported the rate of false-positive and irritant reactions to nickel is higher among children [16], other studies suggest that this high rate might be due to use of adult concentration of allergen for patch testing [17], and the recent series do not confirm this [6]. We could not demonstrate clinical relevance in about half of the cases in our study. In our series nickel sulfate, cobalt chloride, methylisothiazolinone, colophony, potassium dichromate, paraben mix, 4-tert-butylphenon, fragrance mix, thiuram mix, mercapto mix, phenylendiamine base, and formaldehyde were the most common allergens in decreasing order of frequency.

The most commonly reported allergens in a study in Singapore were Nickel (40 %), Thimerosal (15 %), Colophony (9 %), Lanolin (8 %), Cobalt (8 %), Fragrance mix (5 %), and Neomycin (4 %) [18]. While in another study in Turkey, the most commonly documented inciting allergens were Nickel sulfate (46 %), Cobalt chloride (9.5 %), p-Phenylenediamine (9.5 %), Neomycin sulfate 20 % (7 %), Formaldehyde 1 % (4.6 %), Fragrance mix 8 % (3.9 %), CL-methylisothiazolinone 0.01 % (3.1 %), Mercapto mix 2 % (3.1 %), Quaternium 15 % (2.3 %), Benzocaine 5 % (2.3 %), and Potassium dichromate 0.5 % (1.5 %) [19]. In a recent review of five pediatric patch test studies to, the top ten allergens were neomycin, balsam of Peru, fragrance mix, lanolin, cocamidopropylbetaine, formaldehyde, corticosteroids, methylchlorisothiazolinone/methylisothiazolinone, propylene glycol, and benzalkonium chloride [6]. The observed differences in the frequency of the allergens responsible for induction of ACD between the present study and other studies may be explained by a variety of reasons. First and foremost, the prevalence of sensitivity to an individual allergen depends not only on the intrinsic allergenicity of the compound but also on the level of allergen exposure to the population, which may vary from country to country [20–23]. Another important issue is that investigators often employ a variety of test panels and allergen concentrations in different studies, therefore rendering comparisons difficult [23]. There are disagreements as to whether there is seasonal and temporal variation in reactivity to allergens [24]. Moreover, reactivity to some allergens may be influenced by ethnic factors [25].

Gender differences in rates of reactions to a variety of contact allergens have been previously reported [2, 5]. In our study females were significantly more likely to have positive tests. This finding is consistent with previously published studies [5, 12, 15]. Nevertheless, in one study no difference between sex and reactivity to the applied allergens was observed [26].

In the current study, nickel sensitivity was also found to be more frequent in females; however, without statistical significance (Table 4). Ear piercing has been considered as the most common cause of nickel sensitization and the reason for its higher rate in females, with the risk of nickel allergy rising with the number of piercings [12]. Piercing is a common tradition in Iran and is usually performed in girls early in life often followed by long term wearing of golden earing to keep the hole open. Low-carat gold may contain nickel [19] In contrast to studies reporting more potassium dichromate reactivity in adult males, we found no male predominance for this allergen [27, 28].

As previously reported, co-reactivity between cobalt and nickel allergy was observed in our study [2, 18]. We found that in 45.5 % of patients with positive cobalt responses nickel reactivity was also present, while 23.8 % of patients with positive patch tests to nickel also had positive reaction to cobalt. In agreement with our results, Rystedt reported that nickel sensitivity predisposed the patients to cobalt sensitivity [29].

Our results showed that older children tend to show more positive reactions to allergens. This finding is in concordance with a pervious study that showed the rate of patch test positivity was higher in older age groups [12]. Moreover, it should be taken into account that contact dermatitis increases with age and is more common in older individuals [30]. However, according to some researchers an age-dependent decrease in delayed type hypersensitivity may occur with age [30].

In the present study 44.0 % of patients had a personal history of atopy. The relationship between atopy and ACD remains controversial [31, 32]. Although it has been assumed that atopy could be a predisposing factor for the development of ACD, and more reactivity to specific allergens have been reported in atopic patient [33–35]; we found no significant association between personal or family history of atopy and patch test results. In concordance with our findings, some studies indicate that there is a similar prevalence of ACD in individuals with and without atopic diathesis [31, 36]. Hands were the most frequent sites of ACD in our study. Metal preservative and rubber are the most common causes for ACD of this region [11]. Also, in our study metal was the most common causative allergens of ACD.

In the present study, the face was the second most frequent ACD anatomic site. In some studies, the face was the most common site of ACD in children and adolescents [11].

Positive clinical relevance of the positive reactions was considered if the patient described a current or past cutaneous exposure to a product known to contain the allergen to which the patient reacted [7]. For some allergens in the pediatric patient group evaluation of relevance was not possible due to unknown history of exposure.

Methylisothiazolinone is a common preservative found in many cosmetic and toiletry products marketed to both children and adults. It is increasingly known to cause ACD, especially in perioral and perineal regions due to facial or baby wipes [37]. Colophony is a cause of ACD to adhesives and tapes. However, the clinical relevance of a positive patch-test reaction to colophony is often difficult to evaluate [38].

Our study is limited by small sample size, also we were unable to evaluate the relevance for positive patch test for all antigens. Studies with greater sample size and with adequate antigen relevance determination is recommended in Iranian children with ACD.

## Conclusion

Our results indicate that nickel sulfate, cobalt, methylisothiazolinone, and colophony are the most common allergens responsible for induction of allergic contact dermatitis in Iranian children and adolescents. Females tend to show more positive reactions to allergens. These findings are crucial in the treatment, long term management, and proper education of children with allergic contact dermatitis.

## Abbreviation

ACD, allergic contact dermatitis

## Additional files

Additional file 1: Data set on gender, and presence of personal or family history of atopy. (SAV 2 kb) Additional file 2: Data set on age, gender, type of allergen, location of contact dermatitis. (XLSX 20 kb)
